# Advances and Challenges in Pharmacokinetic Modeling for PET Imaging: Compartment Models, Input Functions, and Quantitative Techniques

**DOI:** 10.3390/tomography12050063

**Published:** 2026-04-28

**Authors:** James Hao Wang, Meltem Uyanik, Xue Li, Weijie Chen, Zhijin He, Caitlin Randell, Alan McMillan

**Affiliations:** 1 Department of Radiology, University of Wisconsin School of Medicine and Public Health, Madison, WI 53705, USAabmcmillan@wisc.edu (A.M.); 2 Department of Medical Physics, University of Wisconsin School of Medicine and Public Health, Madison, WI 53705, USA

**Keywords:** pharmacokinetic modeling, positron emission tomography (PET), tracer kinetics, compartmental modeling, quantitative PET imaging, arterial input function (AIF), dynamic PET imaging

## Abstract

Pharmacokinetic modeling in Positron Emission Tomography (PET) imaging has become a cornerstone in cancer research, offering insights into tumor development and progression. These models facilitate the quantification of radiotracer distribution and metabolism, enabling precise measurement of physiological parameters essential for cancer diagnosis, staging, and treatment monitoring. However, accurate pharmacokinetic modeling depends on reliable input function acquisition and partial volume correction techniques to minimize biases in quantitative PET metrics. This review provides a comprehensive overview of current methodologies and advancements in pharmacokinetic modeling for PET oncology imaging. We discuss techniques for acquiring input functions, including arterial, venous, and image-derived input functions (IDIFs), along with population-based input functions (PBIFs). Their strengths, limitations, and clinical applications are critically evaluated. Additionally, we examine quantitative methods such as partial volume correction (PVC) that mitigate the spatial resolution limitations of PET, improving radiotracer quantification in small or heterogeneous tumors. Furthermore, we explore advanced kinetic modeling techniques, including compartmental models, graphical approaches, and data-driven methods, highlighting recent innovations such as machine learning and Bayesian modeling. Key areas for future research in PET pharmacokinetic modeling include integrating hybrid imaging modalities, developing robust patient-specific input functions, and leveraging machine learning to streamline modeling processes. These advancements aim to enhance the precision and clinical utility of PET imaging in oncology, leading to more personalized cancer treatment strategies.

## Simple Summary

Pharmacokinetic modeling is an important tool used with Positron Emission Tomography imaging to understand how a radiotracer travels through the human body. This review explains the fundamental mathematical models driving this process and highlights existing frameworks, methods, advances, and challenges. With new innovations, especially in the area of artificial intelligence, these computationally intensive models are becoming faster and more reliable, enabling further clinical research.

## 1. Introduction

Pharmacokinetic (PK) modeling is a critical method for analyzing the absorption, distribution, metabolism, and excretion (ADME) of any compounds in biological systems. Fundamentally, it is a mathematical framework that describes how substances move through and interact with the body, whether human or animal. This modeling is central to optimizing drug dosing regimens—ensuring that therapeutic concentrations are achieved while minimizing toxicity. Inadequate dosing can lead to subtherapeutic effects or severe adverse reactions, driving the parallel development of pharmacodynamic (PD) modeling with a focus on understanding the biological responses elicited by these compounds [1,2]. Together, PK/PD models utilize compartmental systems to represent physiological regions and simplify complex in vivo processes into tractable and interpretable systems. The complexity of these models can vary significantly depending on the biological target, tracer, or drug characteristics, as well as the scientific or clinical questions at hand.

While PK/PD modeling has historically played a central role in drug development and pharmaceutical toxicology [3], its application has expanded into the field of medical imaging—most notably, positron emission tomography (PET) [4]. PET is a molecular imaging modality that uses positron-emitting radiotracers (radiopharmaceuticals) to noninvasively visualize biochemical processes in vivo, with wide-ranging applications in oncology, neurology, and cardiology [5]. The application of PK modeling to PET imaging stems from the fundamental need to quantify radiotracer dynamics. Hence, in the realm of PET imaging, instead of tracking therapeutic drugs, PK PET models focus on radiotracers and their flow inside the human body.

**Figure 1:**
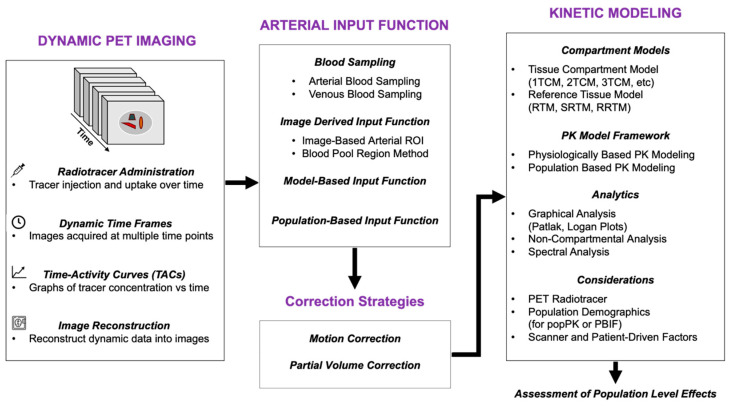
Dynamic PET Imaging Workflow and Analysis Methods. The figure outlines the workflow for dynamic PET imaging, starting from radiotracer administration, image acquisition, and reconstruction, leading to arterial input function estimation. This is followed up by a list of options a user would look for to set up a pharmacokinetic model.

This review aims to provide a comprehensive and structured overview of pharmacokinetic modeling as applied to PET imaging. We begin by introducing core concepts in PK modeling and its broader applications beyond imaging. We then detail the specific modeling frameworks used in PET, including input function estimation methods, compartmental modeling strategies, and various approaches for data analysis. Special attention is given to the logical workflow when implementing a PK model following the structure provided in Figure 1. Finally, we examine emerging methodologies seen in the realm of PK modeling and evaluate ongoing challenges in the field. Through this review, we aim to clarify the methodological foundations, highlight recent innovations, and identify promising directions for future research and clinical translation in PET pharmacokinetics.

## 2. Foundation of Pharmacokinetic Modeling in PET

Implementing a PK model requires a well-defined biological system and a clear understanding of the target organs. While traditional pharmacology models the absorption and elimination of diverse therapeutic drug classes, PET imaging utilizes a highly specific class of compounds: positron-emitting radiopharmaceuticals. Rather than evaluating therapeutic efficacy, PET categorizes these tracers based on their physiological targets, ranging from broad metabolic analogs to highly specific neuroreceptor antagonists. Because of this, the modeling focus shifts heavily to tracking tracer distribution and clearance. Enabling modeling of these tracers reaching and exposing the location of tumors. To mathematically capture these in vivo dynamics, PET PK models rely on two fundamental components: compartments and input functions. Although compartments remain the standard foundation for kinetic modeling, there have been many developments and variations seen in use.

### 2.1. Classical Compartment Modeling

Quantitative modeling in PET imaging plays a critical role in converting dynamic tracer data into meaningful physiological and biochemical parameters. These models generate parametric maps, which are spatially resolved images showing kinetic parameters like distribution volume ($V_{d}$), binding potential (BP), and rate constants (e.g., $k_{1}$, $k_{2}$, $k_{3}$, $k_{4}$). These outputs provide essential insights into tissue function, aiding in the diagnosis and study of diseases by quantifying processes such as metabolism, receptor availability, and enzyme activity. This is often achieved using classical compartment models. While traditionally used to evaluate the full spectrum of drug ADME, their application in PET imaging requires a distinct conceptual shift. Because radiotracers are almost exclusively administered intravenously, the systemic absorption phase is effectively bypassed and becomes irrelevant to the kinetic model. Consequently, distribution emerges as the primary focus in tracking how and where tracers are delivered. These models divide the body into compartments representing different physiological or anatomical regions and mathematically describe the movement of radiotracers between these compartments. The choice of a compartmental model is crucial in accurately representing the distribution of substances over time in various tissues. The differences between compartment models generally arise from differences in complexity and the number of assumed anatomical regions pertinent to the specific tracer.

A fundamental component of any compartment model is the input function, which describes how the tracer is delivered to the tissue of interest. In some contexts, this can be modeled simply. For instance, the two-compartment exchange model (2CXM) defines a central and a peripheral compartment, with the tracer dose administered directly into the central compartment as either an idealized bolus or a constant-rate infusion. While this is effective in fields like Dynamic Contrast-Enhanced MRI (DCE-MRI), where it is often compared with the classical and extended Tofts models, this approach has limitations for PET [6,7]. The actual concentration of radiotracer available to the tissue, as delivered by arterial blood, follows a complex time course that is not captured by a simple bolus or infusion model. To accurately describe the tracer’s dynamic delivery and its effect on the tissue time–activity curve, PET pharmacokinetic modeling generally requires a more precise characterization of the tracer concentration in the blood over time. This is achieved in multiple ways, most commonly by using a measured arterial input function (AIF), which represents the time-course of the parent radiotracer in arterial plasma. This measured function then serves as the input driving the tracer into the tissue compartments.

The simplest model in PET to utilize an AIF is the one-tissue compartment model (1TCM) [8]. In this model, the only considerations are the two rate constants: tracer delivery and elimination via blood flow and metabolism/clearance, respectively. The governing equation is:

(1) $$\frac{dC_{1}}{dt}=k_{1}C_{p}-k_{2}C_{1}$$

In this equation, $C_{1}$ is the tracer concentration in the tissue compartment, and $C_{p}$ is the parent compound’s concentration in plasma, which is usually pre-measured as an AIF. The parameters $k_{1}$ and $k_{2}$ represent the rate constants for uptake and clearance, respectively. The simplicity of 1TCM is the main driver making this model appealing and accessible to many researchers and users. However, its simplicity is also a limitation, resulting in an oversimplified system that may not accurately reflect actual measurements. This model inherently finds most use with simple tracers that do not bind to receptors or undergo complex metabolic pathways.

To account for more complex biological processes, an additional compartment can be added to the 1TCM, producing a two-tissue compartment model (2TCM) [9,10]. The flexibility of these standard compartment models allows users to add as many compartments and connections as needed to model complex kinetic behaviors. However, each added compartment comes with an exponentially increasing computational cost. The 2TCM is expressed with the following equations:

(2) $$\frac{dC_{1}}{dt}=k_{1}C_{P}-\left(k_{2}+k_{3}\right)C_{1}+k_{4}C_{2}$$

(3) $$\frac{dC_{2}}{dt}=k_{3}\cdot C_{1}-k_{4}\cdot C_{2}$$

The three-tissue compartment (3TCM), on the other hand, is based on these equations (option 1):

(4) $$\frac{dC_{1}}{dt}=k_{1}C_{P}-\left(k_{2}+k_{3}+k_{5}\right)C_{1}+k_{4}C_{2}+k_{6}C_{3}$$

(5) $$\frac{dC_{2}}{dt}=k_{3}C_{1}-k_{4}C_{2}$$

(6) $$\frac{dC_{3}}{dt}=k_{5}C_{1}-k_{6}C_{3}$$

**Figure 2:**
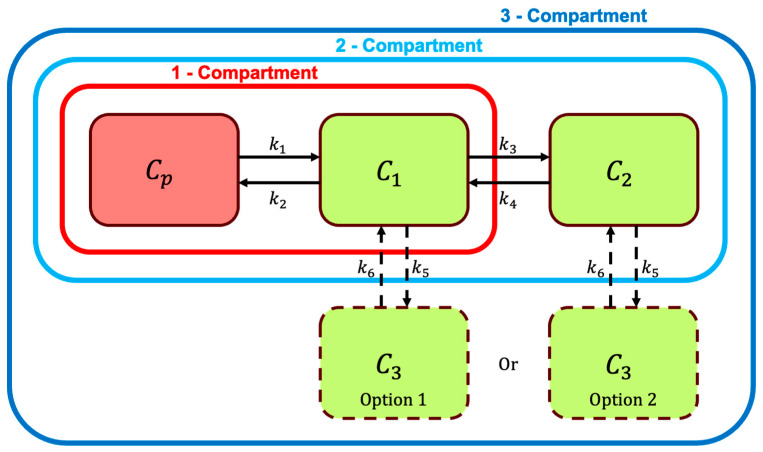
Schematic illustration of one-, two-, and three-compartment models used in pharmacokinetic analysis. The one-compartment model (outlined in red) consists of a single compartment $C_{1}$ representing the tissue of interest, with rate constants $k_{1}$ and $k_{2}$ governing the exchange between plasma $C_{p}$ and the tissue compartment. The two-compartment model (outlined in light blue) adds a second compartment $C_{2}$ representing peripheral tissues, with additional rate constants $k_{3}$ and $k_{4}$ defining the movement between compartments. The three-compartment model (outlined in dark blue) introduces a third compartment $C_{3}$ showing two options of connections, allowing for more complex interactions and physiological processes to be captured with rate constants $k_{5}$ and $k_{6}$.

The equations provided above match setup option 1 in Figure 2. The sequential increase in additional compartments, as demonstrated, substantially increases the number of variables and, therefore, the computational cost to run the PK model. The added complexity allows users to model more complex behaviors. One consideration that also needs to be addressed is the difference in compartment setups; three or more tissue compartments can have different connections between compartments and produce vastly different PK modeling results. Connections between compartments not only can be arranged differently, but can also have additional connections between compartments. An example of this complexity can also be seen in Figure 2 as option 2 for a 3TCM, with the third compartment being connected to the second compartment instead of the first compartment. The equations for this 3TCM setup (option 2) as alternatives to Equations (4)–(6) are:

(7) $$\frac{dC_{1}}{dt}=k_{1}C_{P}-\left(k_{2}+k_{3}\right)C_{1}+k_{4}C_{2}$$

(8) $$\frac{dC_{2}}{dt}=k_{3}C_{1}-\left(k_{4}+k_{5}\right)C_{2}+k_{6}C_{3}$$

(9) $$\frac{dC_{3}}{dt}=k_{5}C_{2}-k_{6}C_{3}$$

As such, the number of compartments is not the only consideration required here; the biological implications driving the connections to be included in the model must also be taken into account. Connections between compartments can be eliminated if the biological pathway does not exist, or there is an intrinsic barrier blocking the flow of the PET radiotracer of interest. For example, compartments can be defined as “irreversible”, resulting in a one-way connection to those compartments.

While it may seem intuitive to employ the most complex compartmental model to capture all physiological nuances, doing so is generally precluded by statistical constraints, such as the risk of overfitting and parameter unidentifiability. Therefore, adopting a more parsimonious approach is both more practical and computationally feasible. The selection of the proper model is ultimately governed by tracer kinetics, tissue behavior, and clinical constraints. For example, radiotracers with rapid kinetics, simpler models with fewer compartments, such as the 1TCM, are typically sufficient. However, certain biological and biochemical complexities inevitably demand the inclusion of additional compartments to prevent inaccurate parameter estimation. For instance, if a radiotracer like ^18^F-FDG undergoes peripheral metabolism in the liver, and the resulting radiolabeled metabolites enter the target tissue, the PET scanner will record a composite signal of both molecules. In such cases, an additional tissue compartment (e.g., expanding to a three-tissue compartment model) is often required to mathematically separate the kinetics of the parent tracer from its radioactive metabolites. Similarly, complex molecular interactions, such as the intracellular internalization of a receptor–ligand complex or a tracer that binds to multiple receptor subtypes with distinctly different affinities, introduce secondary kinetic states that cannot be accurately lumped into a single ‘bound’ pool. Furthermore, highly heterogeneous target regions, such as large tumor volumes exhibiting diverse areas of perfusion and necrosis, may require multi-compartmental structures to account for parallel kinetic pathways. Ultimately, a goodness-of-fit can be used to test the validity and accuracy of these models and see which is more appropriate.

### 2.2. Reference Tissue Models

For most of the tissue compartment models described above, an input function is necessary to define the PET radiotracer’s concentration over time. However, one way to bypass this is by using a reference tissue model (RTM), more commonly utilized in the form of a simplified reference tissue model (SRTM). RTMs effectively act as an extension of the standard compartment models and forego time-consuming measurements via arterial cannulation. Instead, in a standard RTM, the TAC of the reference region is used to model four parameters: $R_{1}$, BP, $k_{2}$, and $k_{3}$. SRTMs assume that kinetics in all regions are fast and simple, allowing for the condensation of the number of parameters to three: $R_{1}$, BP, and $k_{2}$ or $k_{2}^{\prime }$. RTMs and SRTMs usually only apply to quantification with reversible binding. As such, a reduced reference tissue model (RRTM) has been developed and used for these cases of irreversible uptake [8]. The RRTM effectively is a special case of the standard RTM, but with one of its kinetic parameters ($k_{4}$) set to 0 due to the irreversible uptake.

### 2.3. Defining Compartments in a Compartment Model

Under traditional kinetic modeling setups, the multiple compartments in the model do not necessarily translate to different organs. Often in simpler models, such as those with one, two, or three compartments, biological regions can be simplified down to represent multiple organs based on the observed general function. For example, models can be seen combining muscle and fat as absorbers of a radiotracer. An alternative bottom-up approach is to parameterize based on known physiology with compartments assigned to different organs and tissues. This physiologically based pharmacokinetic (PBPK) modeling functions similarly to classical PK modeling, providing the same ADME parameters. However, PBPK provides the advantage of a mechanistic framework using in vitro-in vivo extrapolation to predict profiles of tissue concentrations over time. The PBPK framework has had a long history of use, going as far back as 1937 [11]. It is able to provide users a way to incorporate physio-chemical properties of the tracer and specific physiological differences that may stem from trial design or through patient demographics.

While PBPK models operate with a bottom-up approach, defining compartments based on physiological tissue or organs, population-based pharmacokinetic (popPK) modeling approaches this with a top-down approach. PopPK modeling is empirically driven and uses available pharmacokinetic information to form a model that fits the data [12]. The generation of popPK modeling starts with a simple model defined similarly to a 2CXM with a central and a peripheral compartment, with additional components added one at a time and tested for statistical significance. While compartments in popPK models may not be defined based on anatomical components, they may correspond greatly to actual anatomical processes. PopPK models can account for multiple intrinsic (i.e., age, weight, gender) and extrinsic factors (i.e., drug–drug interactions, diet, lifestyle). Through tracking each of these factors over a large population set, one can identify which ones are more influential and could require dosage adjustments.

### 2.4. Reversible and Irreversible Binding

A fundamental distinction in pharmacokinetic modeling is the nature of the interaction between the radiotracer and its biological target. This interaction is broadly categorized as either irreversible or reversible binding. This categorization dictates the structure of the compartmental model, thereby affecting any derived calculations and parameters from the resulting model. Under irreversible binding, tracers that enter a tissue compartment undergo a transformation, typically enzymatic, which prevents them from leaving. As such, the dissociation rate constant can be defined as effectively zero or negligible within the defined scan duration; many irreversible tracers actually have a slow, negligible clearance that would require a much larger time scale in order to observe a significant change. A prominent example of this type of binding is ^18^F-FDG. When entering the cell through glucose transporters, FDG is phosphorylated into FDG-6-phosphate, becoming metabolically trapped [13]. The implementation of this into a model is mathematically simple. This can be achieved by eliminating the return pathway, resulting in “one-way” pathways between compartments. Reversible binding, on the other hand, occurs when a tracer binds to a target but maintains a significant probability of dissociating and returning to a prior compartment. In these systems, the tracer often reaches a state of equilibrium between compartments. Since the tracer can dissociate, the rate constant is non-zero and important to include in the model.

### 2.5. PK Compartment Summary

Compartment modeling specifically for use with PET data is generally very simple and modular, with adjustments made to each model being made based on each circumstance. This involves a detailed analysis of the tracer, evaluating if the target is able to equilibrate rapidly and achieve well-mixed concentrations. The setup of the compartment model itself has many configurations, requiring users to determine whether PBPK or popPK is better suited for the analysis. Ultimately, a well-defined set of assumptions is necessary to create an accurate model that accurately reflects reality.

## 3. Input Functions

Pharmacokinetic modeling in PET imaging heavily relies on the accurate representation of the input function, which tracks the concentration of a radiotracer in the bloodstream over time. The input function plays a crucial role in determining the parameters of kinetic models, which, in turn, simulate the radiotracer’s interaction with tissues [14,15]. However, inaccuracies in the input function as a result of bias, variance, or noise can significantly impact the accuracy of PET pharmacokinetic models and propagate downstream in their outcomes. Thus, understanding the methods to acquire precise input functions is essential for reliable pharmacokinetic modeling. In the following sections, we will detail the primary methods that are used as input functions, including blood sampling and image-derived input functions. A comparison of these methods can also be found in Table 1.

### 3.1. Blood Sampling-Based Input Functions

Of all the input functions available, blood sampling is considered the gold standard. As the name implies, a direct measurement is taken through blood sampling to derive the input function. The specific methods used to get this measurement can vary, but the core principle is generally the same; blood samples are acquired at pre-determined time points post-injection of the radiotracer. As an invasive method, this can introduce additional risks of complications from catheter placements, which can present as errors in kinetic parameter estimation.

To analyze these blood samples, there are several devices and methods available. For PET radiotracers, the most common method uses gamma counters to measure the radioactivity concentration present in the obtained blood sample. However, other devices have also been used, such as high-performance liquid chromatography (HPLC) and thin-layer chromatography (TLC), to additionally correct for radiochemical purity discrepancies stemming from parent compounds [16].

Under the umbrella of “blood sampling-based input functions,” there are two main approaches: arterial and venous blood sampling [17,18]. These simply dictate the location at which sampling occurs, being either the arteries or the veins. Between them, arterial blood sampling is widely regarded as the most accurate method for measuring the AIF in PET studies. Arterial blood is commonly collected from radial, femoral, or brachial arteries. The first choice is the radial artery because it is superficial and can reduce the risk of bleeding and complications. However, since it is small, it requires extensive skill. The femoral and brachial arteries are also common locations, but may be harder to locate since they are less superficial and are surrounded by other biological structures that could be damaged with improper technique.

Venous sampling operates on a similar principle to the arterial version. Instead of having blood acquired from an artery through a catheter, blood is acquired from a vein through an intravenous cannula [19]. While arterial plasma concentrations are independent of the site of blood sampling, venous concentrations are dependent on the clearance of the radiotracer. Hence, venous blood sampling is dependent on the location [20]. The antecubital vein is the most common location for venous blood sampling. This process is generally considered less invasive and not as technically demanding as arterial sampling. The data acquired will come with some limitations, mainly stemming from the intrinsic arterial–venous difference [21]. Discrepancies between venous and arterial blood concentrations can lead to bias and higher variations resulting from using venous sampling. This can be mitigated by achieving higher blood flow, such as warming the hand to 44°C [22]. The method effectively creates arterialized venous blood, which effectively reduces the arterial–venous difference, making the venous blood characteristics closer to those of the arterial blood. It should be noted that differences in concentrations will still exist, but are lowered so that it can be used as a surrogate for arterial blood.

### 3.2. Image Derived Input Functions

A non-invasive alternative to arterial sampling is the image-derived input function (IDIF). While it is an elegant solution, it comes with some challenges that make it rarely used. This technique fundamentally operates by estimating the AIF directly from PET images. Three primary steps are needed in order to obtain the IDIF: (1) coregister anatomical images to segment arteries, (2) estimate the time–activity curve and correct for partial volume effects, and (3) separate concentrations of parent radioligands from radiometabolites [16]. The third step requires prior knowledge or in vitro analysis of blood samples to determine the estimated percentage of photons emitted belonging to the parent compound. The external blood analysis is necessary because IDIF measures total radioactivity and cannot inherently distinguish the original injected radiotracer from its radioactive metabolic derivatives present in the blood. The combination of steps one and two illustrates the need for accurate region of interest (ROI) placement. Common locations used as ROIs include the carotids, superior sagittal sinus, and blood-pooling regions. Even with accurately placed ROIs, motion, partial volume, and spillover from adjacent tissues can affect the accuracy of the derived TAC. Correction techniques are often employed to adjust for these factors. In a simulation study, results showed that at least 92% of the true intensity could be recovered for an IDIF after applying partial volume correction [23].

### 3.3. Model-Based Input Functions

While AIF can be derived directly or indirectly through blood sampling, model-based input functions (MBIFs) derive the AIF through “reverse engineering”. This method relies on the assumption that the input function is common in all tissue regions within an image [24]. As such, the data acquired can be used with the simultaneous estimation method (SIME) to solve for the AIF [25]. This approach treats the input function as an unknown parameter that can be estimated rather than measured. By modeling the time–activity curves of multiple tissue regions simultaneously, the single common input curve can be derived, which best explains the data across all regions. This method can be used in conjunction with an IDIF to correct for an underestimated AIF.

### 3.4. Population-Based Input Functions

Population-based input functions (PBIFs) provide an alternative approach to all the patient-specific input functions mentioned above. PBIFs, also known as standard arterial input functions, assume that the input curve can be scaled based on the pharmaceutical and population-based parameters, such as injection dose and patient mass [26,27]. Through a set of AIFs measured from a reference population, a radiotracer-specific PBIF is constructed and can then be used for PK modeling. The main draw of using this input function is that it is non-invasive and eliminates the need for continuous arterial cannulation. It is also independent of any imaging noise that would otherwise be present in the IDIF or MBIF. However, this method may be vulnerable to producing inaccuracies in cases of patient-specific variations [28].

## 4. Pharmacokinetic Analysis

Once the appropriate compartment model and input function are selected, the primary focus of the pharmacokinetic modeling shifts towards the quantitative analysis of data. This stage serves as a bridge between the raw TACs and obtaining meaningful physiological indices. Compartment models operate using a mix of variables, including compartment or input concentrations, time, rate constants, and volume. It is through tracking these variables and calculations via PK modeling that we can arrive at commonly reported values: area under the curve, mean residence time, mean transit time, clearance, and steady-state volume of distribution. For PK modeling in PET, these parameters expand to include standardized uptake value (SUV), tissue ratio, and fractional uptake rate (FUR). All of these listed parameters serve as a quantitative framework for characterizing the biological processes of ADME. However, the estimation of these values is not always purely algebraic; this often relies heavily on specific statistical assumptions. These can include noise distributions, system complexity, and initial conditions for each parameter, like independence. As such, it is imperative to understand the various methods that have been developed for interpreting or simplifying the analytical process for PK modeling.

### 4.1. Statistical Frameworks

The choice of a statistical framework for parameter estimation fundamentally dictates how the model interprets noise and uncertainty within the data. The parameters can arise from within the data or from the patients themselves. While the objective remains consistent between statistical methods, the underlying mathematical assumptions and groundwork may differ significantly. The most straightforward approach is to implement a least squares regression operating on a geometric principle. This standard method seeks to minimize the squared difference between model prediction and measured data. While computationally efficient and easy to implement, this standard regression assumes that the measurement error is uniform. As such, weighted nonlinear least squares is more often used in PK modeling in PET. This is to account for non-uniform Poisson-distributed noise present in the raw data. To rigorously compare different models, investigators can employ the Akaike Information Criterion (AIC) or Bayesian Information Criterion (BIC) as goodness-of-fit metrics for validity testing [29]. These methods ultimately penalize overfitting and unnecessary complexity present in the predicted fit.

Moving beyond analysis of individual voxels or subjects, modeling can also be conducted on the population level. Variables such as age, weight, genetic markers, or disease states can be overlooked when performing a standard regression. These variables and others that are not accounted for are fixed or random effects that may cause variations in the raw data and, therefore, skew the model across a population. As such, nonlinear mixed effects (NLME) modeling can be employed, allowing for robust estimation of these effects and accounting for inter-subject variability [30]. This process can be taken one step further and be implemented with a Bayesian framework [31]. This shifts the calculations from fixed parameters to probability distributions, fundamentally setting every parameter as a range of probable values rather than a single number with a confidence interval. Implementing Bayesian inference for highly structured hierarchical models may require the use of Monte Carlo methods.

### 4.2. Graphical Analysis

Multiple-time graphical analysis (MTGA) techniques represent a cornerstone of PET pharmacokinetic modeling, offering a computationally efficient alternative to iterative non-linear fitting [32]. The basic principle of MTGA is that the concentration curves of tissue ROIs and arterial plasma can be combined into a single curve and fitted for the linear phase. Utilizing the slope of this fitting would produce the volume of distribution. The two most common methods, Patlak and Logan plots, largely distinguish themselves by defining the radiotracer as irreversible or reversible.

**Figure 3:**
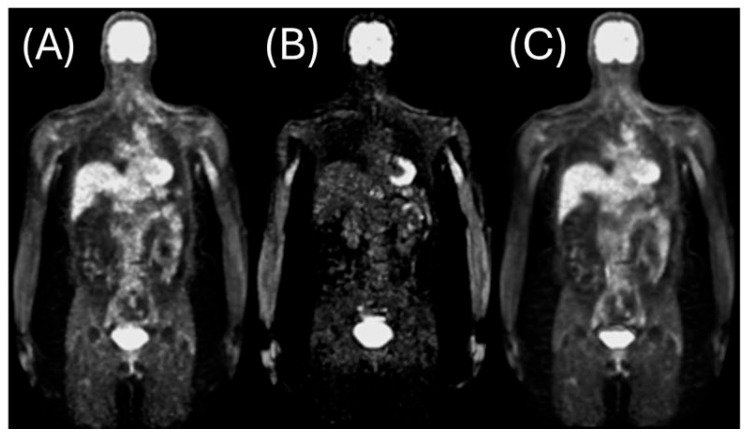
Application of the Patlak plot with a relative starting time of 30 min in estimating the influx rate and distribution volume of tracers in quantitative whole-body dynamic (QWBD) PET imaging. (**A**) Baseline reconstructed image of tracer distribution. (**B**) Estimated influx rate ($k_{i}$) derived from the slope of the Patlak plot, highlighting regions with high influx rates. (**C**) Distribution volume ($V_{d}$) image obtained from the intercept of the Patlak plot, demonstrating improved image quality and noise reduction when advanced reconstruction algorithms are applied.

The Patlak plot is specifically derived for tracers that undergo irreversible binding or uptake [33]. For example, this method is often used with tracers such as ^18^F-FDG. The critical assumption here is that the dissociation rate for at least one compartment is effectively zero. Under this condition, the model assumes that after an initial equilibration period, the concentrations of tracer in plasma and reversible tissue compartments are in equilibrium. As such, the operational equation for the Patlak plot is [34]:

(10) $$\frac{C_{ROI}\left(T\right)}{C_{p}\left(T\right)}=K_{i}\times \frac{\int_{0}^{T}C_{P}\left(t\right)dt}{C_{P}\left(T\right)}+Int$$

where t represents the time after tracer injection, $C_{p}$ is the concentration of tracer in a region of interest, $C_{p}$ is the concentration of tracer in plasma or blood, $K_{i}$ is the clearance determining the rate of entry into the peripheral (irreversible) compartment, and $V_{0}$ is the distribution volume of the tracer. An example of implementing the Patlak plot for analysis can be seen below in Figure 3.

The Logan plot, unlike the Patlak plot, does not fundamentally assume irreversibility. So, all compartments will be considered as reversible, and the net influx rate will no longer be applicable here. Instead, the variable is replaced with the volume of distribution. If a reference region is available, this can be used to calculate the binding potential using the following equation: $BP_{ND}=\frac{V_{T}}{V_{T}^{ref}}-1$. The operating equation for the Logan plot at steady state is [35]:

(11) $$\frac{\int_{0}^{T}C_{ROI}\left(t\right)dt}{C_{p}\left(T\right)}=V_{T}\times \frac{\int_{0}^{T}C_{P}\left(t\right)dt}{C_{P}\left(T\right)}+Int$$

Here, $V_{T}$ represents the total distribution volume, which includes both free and bound tracers in the tissue. It is particularly useful for analyzing tracers that bind reversibly to receptors or other targets in the tissue. The larger the $V_{T}$, the higher the amount of tracer retained in the tissue relative to the plasma, often indicating stronger binding or uptake by the tissue. This equation can be modified and adjusted when a reference region is used instead of arterial blood sampling. The equation can be adjusted as [36]:

(12) $$\frac{\int_{0}^{T}C_{ROI}\left(t\right)dt}{C_{ROI}\left(T\right)}=\frac{V_{T}}{V_{T}^{ref}}\times \frac{\int_{0}^{T}C_{ref}\left(t\right)dt+\left(\frac{C_{ref}\left(T\right)}{\bar{k_{2}^{\prime }}}\right)}{C_{ROI}\left(T\right)}+Int^{\prime }$$

Here, the average $k_{2}^{\prime }$ requires studies of plasma sampling, but can be omitted.

### 4.3. Non-Compartmental Analysis

While compartmental modeling and graphical analysis rely on specific structural assumptions about the tracer’s behavior, non-compartmental analysis (NCA) offers a model-agnostic alternative that focuses solely on the observable properties of the time–activity curve. NCA functions as a model-independent approach that does not require assumptions about the number of compartments or the specific spatial distribution of the tracer. It assumes only that the system exhibits linear superposition, allowing for the estimation of total tracer availability through integration. It is primarily due to its simplicity that it enables quick PK parameter estimation, but it comes with the limitation that these values do not account for physiological processes. The primary outputs include the area under the curve (AUC), which reflects total tracer exposure, and the mean residence time (MRT), which quantifies the average time a molecule spends in the system. These parameters also serve as the foundation for calculating clearance (CL) and volume of distribution at steady state ($V_{ss}$).

### 4.4. Spectral Analysis

Traditional compartmental modeling requires the investigator to define the model structure. However, cases where there are physical abnormalities, such as cancer, can affect how the model is setup ultimately impact how it performs. As such, situations where there are unaccounted physics or interactions can negatively impact performance. One way to address this issue is to use spectral analysis as a data-driven and model-independent approach to evaluate data [37]. This method assumes that the tissue response is composed of a continuum of possible kinetic frequencies. By evaluating each of the underlying components, the spectral analysis approaches, such as principal component analysis (PCA), can be used to distinguish between tumor and normal tissue kinetics [38]. This can be used down the line for further analysis using NCA or standard compartmental PK modeling [39].

## 5. Recent Developments

PK modeling is a well-established discipline, and its fundamentals have not shifted drastically. However, research developments have been continuously made to evaluate new tracers with PK models or find ways to optimize the model itself. The integration of artificial intelligence (AI) and machine learning (ML) has opened the door to further developments, improving the process for PK modeling, but has yet to find large implementation in PK and PK-PD modeling [40]. At its core, modern AI operates by taking massive datasets and uses them to train a computer model to recognize patterns, rather than relying on human-written programming rules. Deep learning, an advanced subset of AI, relies on artificial neural networks to capture complex, non-linear relationships within high-dimensional data. Despite their predictive power, these models are fundamentally constrained by their reliance on extensive datasets, split between validation, training, and test sets. In the context of PET pharmacokinetic modeling, acquiring massive cohorts of paired ground-truth data, such as gold-standard invasive arterial blood samples, can be logistically challenging. Consequently, highly generalized AI models remain scarce, resulting in tools that are often rigidly tailored to the specific radiotracer and clinical protocol used during their development.

Despite these challenges, we have seen increasing developments using AI with PK modeling. One example utilizes neural networks to make a predictive model to achieve a faster and computationally less intensive modeling approach [41]. The implementation of AI is not limited to just the compartment modeling component but can be applied at multiple stages. The raw PET data acquired can be processed through an AI model for denoising, motion correction, and partial volume correction [42]. AI has also found use cases in PET data segmentation, sometimes used with other imaging modalities, enabling ROI generation, which is particularly useful for generating IDIFs.

Applications for AI directly on PK modeling have found use on two levels: the input function and the PK model. On the input function front, by training deep convolutional neural networks (CNNs) on large cohorts of historical data, AI models can now accurately predict the arterial input function directly from image data, effectively correcting for partial volume effects and reducing the need for invasive arterial cannulation [43]. One application of this found similar results using a deep-learning derived input function (DLIF) to arterial blood sampling tested in small animals [44]. Beyond input functions, deep learning is also being applied directly to parameter estimation, where neural networks learn the non-linear mapping between time–activity curves and kinetic macro-parameters (such as $k_{i}$ or $V_{T}$). While these approaches incur a substantial upfront computational burden during the training process, they offer a highly computationally efficient alternative once the model is deployed.

## 6. General Considerations

### 6.1. Tracer Characteristics

The successful implementation of a pharmacokinetic model is inextricably linked to the physical and chemical properties of the radiotracer employed. While the mathematical framework of a simple PK model like the 2TCM may be theoretically universal, the practical execution is dictated by the radionuclide’s half-life and the tracer’s biological affinity. As the use of PET imaging increases, the number of radiotracers involved has grown to encompass a wide range of options. The most commonly used tracer, ^18^F-FDG, exhibits very slow dissociation kinetics relative to the scan duration; it is effectively “trapped” within the metabolic pathway, necessitating an irreversible model structure [13]. Ga-FAPI, another highly utilized PET radiotracer, has much faster kinetics and is therefore considered reversible [45]. This simple difference exemplifies the importance of knowing key characteristics of your desired tracer of interest and how it will affect the design and analysis of the PK model.

### 6.2. Challenges

A fundamental limitation of standard pharmacokinetic modeling is the necessary simplification of complex biological systems into linear compartmental structures. Most kinetic models assume first-order kinetics, where the rate of transfer is directly proportional to concentration. However, biological reality is frequently non-linear. Processes such as enzyme-mediated metabolism or receptor–ligand binding follow Michaelis–Menten kinetics, where rates can saturate if the concentration of the substrate (or radiotracer mass) approaches the capacity of the system [46]. If the specific activity of the radiotracer is low, or if the target density is altered by disease, the assumption of linearity may be violated, leading to parameter bias. Furthermore, models often assume a “well-mixed” compartment, ignoring intra-voxel heterogeneity where vascular density, necrosis, and active tumor tissue may coexist within a single resolution element [47]. This discrepancy between the idealized mathematical model and the stochastic, heterogeneous nature of in vivo biology remains a significant source of modeling error.

**Figure 4:**
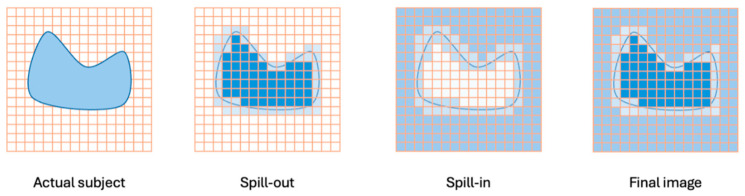
Illustrates the influence of PVE in imaging: the leftmost panel shows the actual subject, the second panel represents spill-out where the signal spreads out of the region of interest, the third panel depicts spill-in from neighboring tissues, and the rightmost panel displays the combined effects in the final image. The result is a loss of signal specificity, making the structure appear larger but less intense. These inaccuracies can lead to significant misinterpretations in tumor characterization, therapy response evaluation, and disease progression monitoring. Consequently, various PVC methods have been developed to minimize these errors and restore the true signal distribution in the region of interest.

Beyond biological complexity, the accuracy of kinetic parameters is strictly bounded by the physical limitations of the imaging itself. PET data may be acquired in unsteady or time-variant conditions, where underlying physiology shifts during the scan. Examples of this state include fluctuations of neurotransmitters such as dopamine or external interventions via pharmacologic dose, the presence of which can lead to unforeseen changes and fluctuations appearing in the data, making analysis much more difficult [48]. In addition to these physiological factors affecting the temporal signal, spatial accuracy is impacted by physical limitations of the imaging hardware itself. One of the most pervasive issues is the partial volume effect (PVE), caused by the limited spatial resolution of PET scanners (typically 3–5 mm). When a target structure is smaller than roughly three times the scanner’s resolution, the signal “spills out” into surrounding tissue, causing a systematic underestimation of activity concentration and, consequently, kinetic rates. Conversely, “spill-in” from adjacent high-activity regions can falsely elevate values in background tissues [49]. Both of these partial volume effects can be seen below in Figure 4. Compounding this is patient motion—ranging from rigid head movements to complex non-rigid respiratory and cardiac cycles. Motion blurs the time–activity curve and causes misalignment between the dynamic PET frames and the CT or MRI anatomical reference, used for attenuation correction and ROI definition. Without rigorous motion correction or gating strategies, the resulting kinetic parameters may reflect movement artifacts rather than true physiology [50]. Hence, this necessitates motion and partial volume correction methods to be applied to the PET data.

Finally, the transition from region-based to voxel-wise modeling introduces substantial computational hurdles. While fitting a model to a single ROI curve is computationally trivial, generating parametric maps requires solving non-linear iterative problems for each individual voxel. Depending on the matrix size of the PET acquisition, the number of individual voxels can be an extremely large number. This process is computationally expensive and mathematically perilous; low-count voxels often have high noise, leading to “noisy” objective functions with multiple local minima. Standard optimization algorithms may fail to converge or may converge to biologically implausible values (e.g., negative rate constants) if not tightly constrained. While advanced methods like linearized solutions (e.g., basis functions) or neural networks (AI) are mitigating these costs, the trade-off between computational speed, algorithmic stability, and quantitative precision remains a central challenge in high-throughput kinetic analysis [41,51].

## 7. Conclusions

Pharmacokinetic modeling transforms PET from a qualitative visualization tool into a quantitative instrument capable of measuring fundamental physiological processes in vivo. As detailed in this review, the accuracy of this quantification relies on a rigorous methodological chain, ranging from the selection of appropriate compartmental structures and statistical frameworks to the precise characterization of the arterial input function. While physical limitations, such as partial volume effects, patient motion, and inherent noise, continue to pose as challenges to parameter accuracy, the field is rapidly evolving to address them through improved correction strategies and computational innovations. The emerging integration of artificial intelligence and machine learning represents a pivotal advancement, offering the potential to automate complex analyses, derive non-invasive input functions, and enhance the robustness of voxel-wise estimation. Ultimately, the continued refinement of these modeling strategies is vital for the future of precision oncology, providing clinicians with the reproducible, quantitative biomarkers necessary to optimize diagnosis, monitor treatment response, and tailor therapeutic interventions to the individual patient.

## Figures and Tables

**Table 1:** Comparison of Input Function Methods.

Input Function	Invasiveness	Accuracy	Complexity	Clinical Feasibility
AIF-Arterial	High: Requires arterial catheterization	Highest: Gold Standard	Moderate: Requires skilled personnel for catheterization	Low: Largely restricted to research settings due to invasiveness
AIF-Venous	Moderate: Samples drawn from peripheral vein	Low/Moderate: Arterial–venous differences	Low: More accessible to obtain blood sample	Moderate: Less invasive but also less accurate
IDIF	Low: Can require samples for calibration	Moderate: Susceptible to partial volume effects	Moderate: Requires precise image processing and correction algorithms	Low: limited implementation of PET radiopharmaceuticals
MBIF	Low: Can require samples for scaling	Moderate: Can be good with a good reference region	High: Requires mathematical modeling and validation	Low: limited implementation of PET radiopharmaceuticals
PBIF	Low: Can require samples for scaling	Moderate: Assumes patient matches population	Low: Once derived, it is simple to apply	High: Easily implemented

## Data Availability

No new data were created or analyzed in this study.

## Abbreviations

The following abbreviations are used in this manuscript:

PET	Positron Emission Tomography
PK	Pharmacokinetic
ROI	Region of Interest
AIF	Arterial Input Function
TAC	Time–Activity Curve
